# The effectiveness of a Supported Self-management task-shifting intervention for adult depression in Vietnam communities: study protocol for a randomized controlled trial

**DOI:** 10.1186/s13063-017-1924-5

**Published:** 2017-05-05

**Authors:** Jill Murphy, Charles H. Goldsmith, Wayne Jones, Pham Thi Oanh, Vu Cong Nguyen

**Affiliations:** 1 Centre for Applied Research in Mental Health and Addictions, Simon Fraser University, Suite 2400, 515 W. Hastings Street, Vancouver, BC V6B 5K3 Canada; 2 Faculty of Health Sciences, Simon Fraser University, Blusson Hall, Room 11300, 8888 University Drive, Burnaby, BC V5A 1S6 Canada; 3 Institute of Population, Health and Development, 18 Lane 132, Hoa Bang, Yen Hoa, Hanoi, 122667 Vietnam

**Keywords:** Supported self-management, Depression, Task-shifting, Vietnam, Psychosocial treatment, Low and middle-income countries, Stepped-wedge design

## Abstract

**Background:**

Depressive disorders are one of the leading causes of disease and disability worldwide. In Vietnam, although epidemiological evidence suggests that depression rates are on par with global averages, services for depression are very limited. In a feasibility study that was implemented from 2013 to 2015, we found that a Supported Self-management (SSM) intervention showed promising results for adults with depression in the community in Vietnam.

This paper describes the Mental Health in Adults and Children: Frugal Innovations (MAC-FI) trial protocol that will assess the effectiveness of the SSM intervention, delivered by primary care and social workers, to community-based populations of adults with depression in eight Vietnamese provinces.

**Methods/design:**

The MAC-FI program will be assessed using a stepped-wedge, randomized controlled trial. Study participants are adults aged 18 years and over in eight provinces of Vietnam. Study participants will be screened at primary care centres and in the community by health and social workers using the Self-reporting Questionnaire-20 (SRQ-20). Patients scoring >7, indicating depression caseness, will be invited to participate in the study in either the SSM intervention group or the enhanced treatment as usual control group. Recruited participants will be further assessed using the World Health Organization’s Disability Assessment Scale (WHODAS 2.0) and the Cut-down, Annoyed, Guilty, Eye-opener (CAGE) Questionnaire for alcohol misuse. Intervention-group participants will receive the SSM intervention, delivered with the support of a social worker or social collaborator, for a period of 2 months. Control- group participants will receive treatment as usual and a leaflet with information about depression. SRQ-20, WHODAS 2.0 and CAGE scores will be taken by blinded outcome assessors at baseline, after 1 month and after 2 months. The primary analysis method will be intention-to-treat.

**Discussion:**

This study has the potential to add to the knowledge base about the effectiveness of a SSM intervention for adult depression that has been validated for the Vietnamese context. This trial will also contribute to the growing body of evidence about the effectiveness of low-cost, task-shifting interventions for use in low-resource settings, where specialist mental health services are often limited.

**Trial registration:**

Retrospectively registered at ClinicalTrials.gov, identifier: NCT03001063. Registered on 20 December 2016.

**Electronic supplementary material:**

The online version of this article (doi:10.1186/s13063-017-1924-5) contains supplementary material, which is available to authorized users.

## Background

Depressive disorders contribute considerably to the global burden of disease, and are expected to become the leading cause of Disability-adjusted Life Years (DALYs) worldwide by 2030 [1]. Despite the high prevalence of depression and its detrimental impact on quality of life, services for depression are limited in much of the world, particularly in low- and middle-income countries (LMICs) [2]. Mental health specialists are often in short supply, with services concentrated in often inaccessible and overburdened tertiary-care facilities [3, 4]. As a response to this treatment gap, task-shifting approaches, whereby nonspecialist providers deliver evidence-based and cost-effective services, have been recommended [5–7].

While epidemiological evidence about the prevalence of common mental disorders in Vietnam is limited, existing studies suggest that prevalence is similar to that found in much of the world, with rates of approximately 20% identified in some studies [8–10]. Mental health specialist services are limited, with the mental health system predominantly addressing psychotic disorders and epilepsy in tertiary-care facilities [3, 11]. Although patients with severe depression might receive pharmacological treatment in tertiary-care facilities, counseling and psychosocial interventions are almost nonexistent [11]. In its National Mental Health Strategy for 2015–2020, the Government of Vietnam has prioritized the enhancement of community-based mental health services including services for people with depression.

Supported Self-management (SSM) for depression is an approach through which providers support people with depression to learn and implement mood management skills [12]. SSM is a low-cost intervention that is consistent with guidelines for chronic illness care [13, 14]. It is appropriate for use in primary care settings by nonspecialist providers [15] and fits with a stepped-care approach that has been recommended for mental health service delivery in both high-income countries [16, 17] and LMICs [6, 18, 19]. In a systematic review, Lucock et al. (2011) [15] found that SSM produces an effect size that is on par with conventional depression treatment.

The SSM approach is fitting for community-based settings in LMICs, as it requires minimal resources, can be implement by primary care or lay providers using a task-shifting approach, and can be adapted to meet local settings. A SSM approach is consistent with the call by the global health community for “frugal innovations” that use few resources to meet pressing challenges [20, 21]. Evidence about the effectiveness of SSM in LMIC settings, however, is needed.

From 2013 to 2015, we conducted a pilot study in Hanoi, Vietnam to test the feasibility of training primary care providers to screen for, and treat, mild-to-moderate depression using SSM. The pilot study also tested the feasibility of conducting a full-scale randomized controlled trial (RCT), including the feasibility of primary care provider and patient recruitment. The pilot study demonstrated proof of concept and the SSM intervention was found to be acceptable and effective for use in Vietnam. At baseline, patient (*n* = 71) scores on the Self-reporting Questionnaire-20 (SRQ-20), a measure of depression symptoms that has been validated in the Vietnamese context [22, 23], varied from 8 to 17 (out of a possible 20) with a mean score of 10.9. Administrative errors led to a failure to obtain routine follow-up of SRQ-20 scores at the planned 2-month time period, but follow-up scores were eventually obtained for all participants (time between scores varied from 62 to 241 days). Upon follow-up, SRQ-20 scores varied from 1 to 13 with a mean score of 4.7, with 62/71 (87%) patients having SRQ-20 scores in the normal range.

**Fig. 1:**
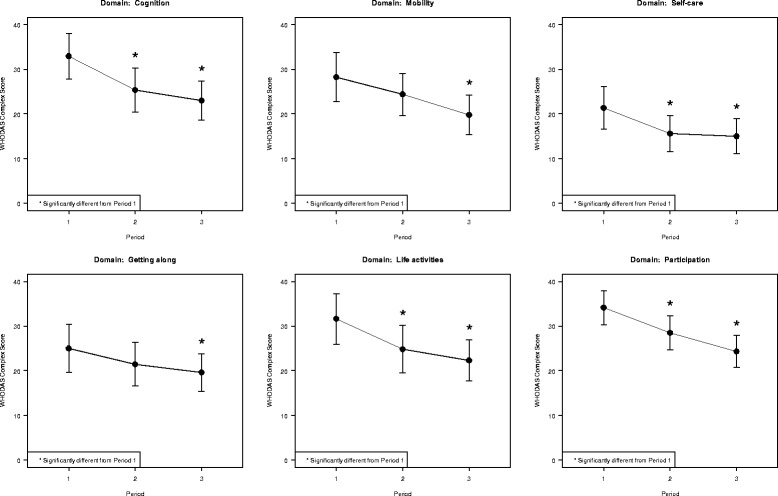
World Health Organization’s Disability Assessment Scale (WHODAS 2.0) domain scores at 0 months and 2 months

The World Health Organization’s Disability Assessment Scale (WHODAS 2.0) [24] is a generic assessment instrument for health and disability across all diseases, including mental, neurological and addictive disorders. Scores are calculated in six separate domains (cognition, mobility, self-care, getting along, life activities and participation) and an overall score is based on the separate domain scores. Over the 2-month course of treatment, repeated measures analysis of variance (ANOVA) of the overall WHODAS 2.0 scores showed that the scores declined statistically significantly with mean values indicating less illness over time. Mean overall scores, which may vary from 36 to 180, dropped from 83.4 at baseline, to 71.9 after 1 month of treatment and 64.7 after 2 months. Separate repeated measures ANOVAs for the scores for the six subscales all showed at least one period with a statistically significant decline from baseline (see Fig. 1 for a plot of the means and 95% CIs for each of the domains).

The results of the feasibility study suggest that SSM might be a viable and effective approach to treating mild-to-moderate depression in Vietnam. Given the shortage of mental health specialist providers and the government priority to improve community-based care for depression, the delivery of SSM through a task-shifting model could contribute substantially to addressing the depression treatment gap in Vietnam. This paper describes the study protocol of an RCT testing the effectiveness of the SSM intervention for treating mild-to-moderate depression in eight Vietnamese provinces. Based on the results of the feasibility study, we hypothesize that SSM would be more effective than treatment as usual in the treatment of mild-to-moderate depression in community-based settings in Vietnam.

## Methods/design

### Study setting

The purpose of this study is to test the effectiveness of the SSM intervention to treat mild-to-moderate depression in Vietnamese community-based populations. The SSM intervention will be tested in eight provinces across Vietnam (see Table 1). The study will take place in two districts of each province, targeting two communes (municipal subdivisions) in each district for a total of 32 communes.

**Table 1:** Study provinces by region

Northern provinces	
Thanh Hoa	
Thai Nguyen	
Quang Ninh	
Central provinces	
Da Nang	
Quang Nam	
Khanh Hoa	
Southern provinces	
Long An	
Ben Tre	

### Design

**Fig. 2:**
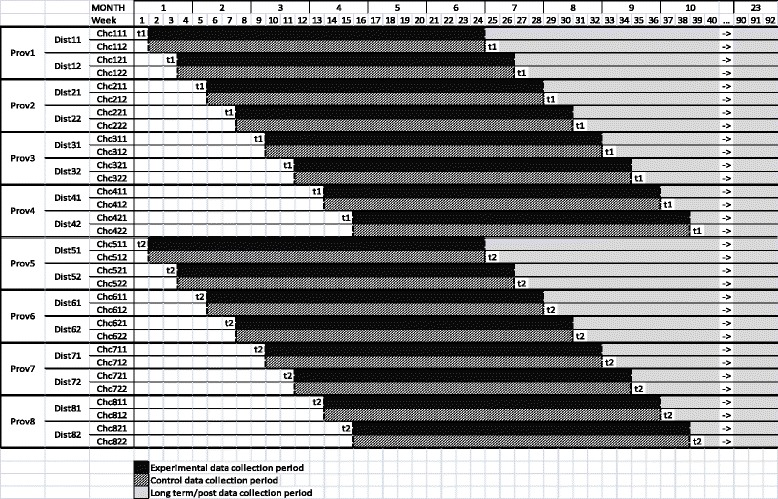
Study design

**Fig. 3:**
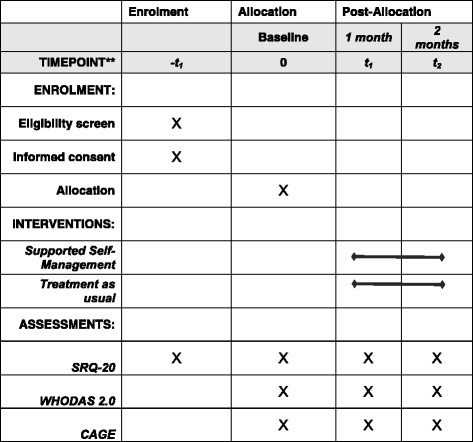
SPIRIT Figure: MAC-FI Study Protocol

The study uses a cluster-randomized, stepped-wedge controlled trial design. Randomization will occur at the commune level and communes will be assigned to receive either the SSM intervention first or the control condition first. The control condition will be enhanced treatment as usual. Those communes assigned to receive the control condition first will receive the SSM intervention 4 months later. This will ensure that the study is conducted ethically and will not deny evidence-based services to members of the control group. Participants in the intervention and control groups will be from different communes, or municipal districts, meaning that they do not access the same service units. This will minimize the risk of contamination. Figure 2 shows how the design will operate. The *black rows* depict the experimental data collection periods, *diagonally striped rows* are the control data collection periods, and the *gray rows* represent the long-term data collection period. The primary measure of interest is the proportion of individuals in the experimental versus the control group (*black* versus *diagonal striped*) that went from being a case of depression (SRQ-20 > 7) to being non-case (SRQ-20 < 8) over the experimental data collection period. Based on the feasibility case rate of a mean of one case every 14 days, each commune is expected to have eight completed cases (baseline, month-1 and month-2 measures) per year for a total *n* of 256 cases. Figure 3 shows a SPIRIT figure depicting the study design. Additional file 1 provides an overview of the elements of the study protocol, as described in a SPIRIT checklist.  Recruitment will continue until the desired sample size is reached and data can be analyzed to assess the effectiveness of the intervention.

### Characteristics of participants

This study will test the effectiveness of a SSM intervention for reducing depression among adults in Vietnam. The study group consists of adult patients over the age of 18 years in 32 communes in eight Vietnamese provinces. Patients will be recruited by primary care providers at Commune Health Stations (CHSs), and by social workers in the community. Primary care providers at CHSs will screen patients during regular consultations, while social workers will screen community members who have been identified as vulnerable (e.g., new mothers, people experiencing bereavement, economic loss). Based on the results of the feasibility study, we do not anticipate major difficulty in recruitment. However, should recruitment prove challenging in some communes, we will expand the study to additional communes and consider expanding the duration of the study to ensure that we reach our target sample size.

### Outcome assessment

Demographic measures for both treatment and control clusters will be collected at baseline and will include: age, sex, education and economic status. The outcome measures will be collected at baseline, at 1 month and at 2 months following the initiation of SSM. The primary outcome measure will be the SRQ-20 Vietnamese version [22]. We selected the SRQ-20 as the most suitable measure following an extensive review of existing measures for depression [25]. The SRQ-20 has been carefully developed for use in Vietnam, it has been adapted to address culturally appropriate manifestations of depression symptomatology and its validity has been tested in studies within Vietnam [23]. As described above, through our feasibility study, we have good information about its performance and utility in Vietnam. We will also use the WHODAS 2.0 as a secondary measure to evaluate changes in functional ability [24]. This aspect of outcome is important as it allows an evaluation of important changes in an individual’s ability to perform activities of daily living and return to social and occupational activities. We have experience using the WHODAS 2.0 in our feasibility study and found that it provided meaningful information about changes in function with treatment. We have also added the Cut-down, Annoyed, Guilty, Eye-opener (CAGE) Questionnaire, a four-item alcohol misuse questionnaire [26] to the WHODAS 2.0, as research suggests that alcohol misuse and depression are related in Vietnam, especially among men [27]. The addition of the CAGE will help us to better understand the extent to which depression and alcohol misuse co-occur. Adverse events will be collected using a form for adverse events at each assessment point and any adverse event related to the intervention will be reported. We experienced no adverse events in our feasibility study.

### Informed consent

Written informed consent will be obtained from participants at recruitment, following initial screening using the SRQ-20 and prior to WHODAS 2.0 and CAGE assessment. Informed consent will be obtained by trained health workers, and a copy of the Information Letter and Consent Forms will be provided to participants.

### Confidentiality

All electronic data will be saved on a secure server. The hard copy questionnaires will be stored in a locked cabinet at the CHS or at the project’s head office in Hanoi. Only authorized research team members will have access to hard copy and electronic questionnaires. The hard copy questionnaires and the electronic versions will be saved for 5 years and will then be destroyed, according to Vietnamese requirements. All prescreening data for patients that are not recruited to the study will be stored in a locked cabinet in the CHS, and will be destroyed after 1 year, as is standard practice in Vietnam.

Study participants will be assigned a unique code. This code will be used in the database to ensure confidentiality. No identifying information, such as name or demographic features, will be attributed to specific participants to uphold confidentiality.

### Inclusion and exclusion criteria

Inclusion criteria for the study are: (1) participant meeting caseness based on an SRQ-20 score of >7 and (2) consent of participant to participate and complete all measures. Exclusion criteria are: (1) cognitive disturbance, (2) symptoms of severe mental illness, including psychosis, severe depression and suicidal ideation, (3) visual or hearing impairment such that patients cannot use the manual or work with a care provider and (4) illiteracy. Patients presenting with symptoms of severe mental illness, including severe depression, will be referred to specialist services at the time of screening.

If patients enrolled in the study should become suicidal or experience psychotic symptoms during the study, primary care providers and social workers will be instructed to refer patients to tertiary care. Patients receiving treatment in tertiary care will no longer remain in the study. Every effort will be made to follow up and conduct outcome measures with these patients; however, the severity of their illness and nature of their treatment may make this difficult.

### Intervention

SSM is an approach to chronic illness management that acknowledges the active role of the patient in their own care, taking a collaborative approach in which a patient is supported by a health care provider, peer or family member [12]. In SSM for depression, patients are given a SSM guide that is based on the principles of cognitive behavioral therapy, including behavioral activation, cognitive restructuring, and problem solving [12]. Patients are provided with encouragement and coaching in using the SSM guide. In systematic reviews of SSM in Western contexts, SSM was found to have an effect size that is similar to conventional treatments for depression, such as psychotherapy, and was shown to be more cost-effective than antidepressants or psychotherapy [12]. SSM is also cost-effective for both patients and health systems and can replace unnecessary prescription of antidepressant medications in patients with mild-to-moderate depression [12]. The SSM intervention that is being tested in Vietnam uses the *Antidepressant Skills Workbook* [28], which was developed by mental health specialists at Simon Fraser University. The *Antidepressant Skills Workbook* was validated for its cultural acceptability in Vietnam and was translated and back translated for semantic equivalence during the pilot study.

The intervention training is a course delivered to primary health care workers, social workers and social collaborators (volunteer lay social workers) about depression and SSM that has been culturally adapted for use in Vietnam. Primary health care staff at CHSs, social workers and social collaborators will be provided with enhanced training about depression, which will supplement their minimal mental health training. This includes information about the symptomatology and etiology of depression and evidence about treatment options, including SSM. This training is informed by research about commune health workers’ existing levels of knowledge, training and explanatory models of common mental disorders [27] to ensure that the training is culturally relevant and responsive to health workers’ training and experience. Health and social workers will subsequently be introduced to, and trained in using, the SRQ-20, WHODAS 2.0 and CAGE to identify depression, disability and alcohol use among patients. They will also be trained in using the adapted version of the *Antidepressant Skills Workbook* and in the SSM approach. Following health worker training, patients will be screened when accessing primary care services at the CHS or in the community and recruited into the study. Patients will be provided with the *Antidepressant Skills Workbook* and will be supported in its use by a social worker or social collaborator for 2 months. SRQ-20 and WHODAS 2.0 measures will be taken at baseline, at 1 month, and at 2 months. The SSM intervention is delivered in a short period of time, often over the course of 9–12 weeks [12]. The feasibility study results showed a pattern over the three data points, with depression and functional ability scores improving from baseline to 2 months.

### Control condition

The control condition will be enhanced treatment as usual. For the most part, depression is not recognized or treated in primary care centres in Vietnam and it is, therefore, likely that patients who are receiving primary care at CHSs or who have been screened by social workers in the community in communes that are randomized to the control condition will receive little or no treatment for depression. Enhanced treatment as usual will consist of the following:

1. Health and social workers will receive training that recommends “watchful waiting,” except in severe cases, including suicidal ideation, psychosis and the possibility of causing harm to others. Training will also include information about how to respond to emergency situations
2. Patients will receive a leaflet adapted from the *Beyond Blue “Understanding Depression”* Leaflet (www.beyondblue.org.au) that provides information about depression, providing patients with information about their diagnosis, including depression symptoms, risks and approaches to treatment

Patients in communes randomized to the control condition will be offered SSM following the period of control data collection, after 4 months. At the time of the 1- and 2-month assessments, we will capture what, if any, additional treatment has been accessed by the patient (e.g., services at the tertiary-care level, informal or formal counseling, supplements, traditional Vietnamese interventions, sleeping pills). Concomitant care is not prohibited during the trial, but the study team will record any formal or informal treatments and services that might be accessed by patients during their enrollment in the study.

### Randomization

Randomization will be done using permuted blocks (to conceal allocation) and will be stratified by district. The randomization sequence will be developed and controlled by an individual (CHG at Simon Fraser University) not otherwise involved in the study to ensure fidelity. Details of the randomization, such as block sizes and software implementation, will be outlined in the Statistical Analysis Plan (SAP) to prevent anticipated allocation of the communes to getting the early or later timing of the SSM intervention. The SAP will be developed after this protocol is submitted for publication; however, the plan is to link them when they are published.

### Blinding

It will not be possible to institute full blinding because of the nature of the intervention. Project outcome assessors will be blinded. Based in the project head office in Hanoi, outcome assessors will conduct the 1- and 2-month assessment interviews with patients by telephone. The outcome assessors will have no information about patients, where they are located or whether they are allocated the intervention or control condition. The project research coordinator will collect telephone numbers from the participants, and will assign them an appointment with the outcome assessor. The outcome assessor will then call the telephone number with no additional information about the participant. Participants will be instructed not to share information about whether they are part of the intervention or control group with outcome assessors to avoid contamination.

### Data collection

Baseline data will be collected by primary care staff, social workers and social collaborators. Outcome assessors will collect follow-up data by telephone at 1 and 2 months. Outcome assessors will receive 3 days of training on the use of the SRQ-20, WHODAS 2.0 and CAGE.

Several strategies are in place to participant retention and to encourage adherence to the study protocols. Patients who participate in visits with social workers every 2 weeks and complete the final outcome evaluation will be provided with a small gift (e.g., hand lotion). The Department of Labor, Invalids and Social Affairs (DOLISA) in each province will also provide a bonus to social workers for their participation in the study.

We will continue to collect data from participants who discontinue their participation in the study by ensuring that we have up-to-date telephone numbers for all participants. As symptoms improve, patients who have been unable to work will likely return to employment. In cases, such as with agricultural laborers, where the nature of their work might prohibit their participation in the study, we will provide additional compensation to these participants to encourage them to complete the outcome assessment.

### Power calculation

The number of subjects required for this randomized cluster trial was calculated by estimating the number that would be required in a completely randomized design and then adjusting this number by the design factor associated with a randomized cluster trial (Table 2). The number of subjects required for a completely randomized design was calculated by the R function pwr.t.test [29] using the following characteristics:

- An effect size of 0.5 (where a small effect size is 0.2; medium is 0.5; large is 0.8)
- Significance level – type 1 error rate (0.05)
- a two-sample design

Using a medium effect size (0.5) results in an estimate of 64 subjects per arm or 128 in total at an 80% power.

**Table 2:** Sensitivity analysis

Effect size	ICC	Number per cluster	Total *N* required
0.5	0.05	8	173
0.8	0.05	8	69
0.2	0.05	8	1063
0.3	0.05	8	474
0.4	0.05	8	268
0.45	0.05	8	212
0.5	0.025	8	150
0.5	0.01	8	132
0.5	0.005	8	132
0.45	0.005	8	163

*ICC* intracluster correlation coefficient

The Design Effect (DE) is estimated by the formula:

$$ D E = 1 + \left(\mathrm{n} - 1\right) \times \mathrm{p} $$

where

*DE* = Design Effect

*n* = number of subjects in each cluster

*p* = ICC (intracluster correlation coefficient)

No estimates of the SRQ-20 intracluster correlation coefficient (ICC) are available; however, within the research literature on estimating sample sizes, the ICC is usually described as small with values of 0.01 to 0.05 – with a median of around 0.01. We have used the upper value of 0.05. This is the number of participants who will be found to have an SRQ-20 > 7 and will be followed up over the 2 months. This was estimated from the feasibility data, which produced approximately one subject every 14 days within primary care centres (or one every 2 weeks). We therefore estimate a mean of eight subjects per commune would be completed in the experimental or control time period, meaning that the total *n* during the study period is 32 clusters × 8 completed subjects per cluster or 256 subjects. The DE associated with eight subjects per cluster, a medium effect size of 0.05, and an ICC of 0.05 is 1.35, meaning that the randomized cluster study requires 128 × 1.35 or 173 subjects which is below the anticipated *n* of 256.

If the expected effect size is under 0.45 the study is likely underpowered. In the feasibility study, nine of the 68 (13%) subjects remained at 8 or higher in the second SRQ-20 – thus, 87% dropped below 8, a very large effect of the intervention. While the effect size in the pilot study would fall into the “large” category the variable follow-up time makes the estimate suspect, and thus we used a medium effect size to account for the expected impact over a 2-month period. Based on the feasibility results of eight patients per cluster, an expected minimum effect size of 0.4, an ICC of no more than 0.05 the proposed study would be powered at approximately 80% with a 0.05 alpha level.

### Analysis

This high-level, statistical-analysis description is meant to give an overview of what will be included in the analysis of the data collected during this trial. A more detailed description of the methodology will be provided in the Statistical Analysis Plan (SAP) [30] which will be written once this protocol has been submitted for publication. The main reasons for this are that the protocol has space restrictions of the details needed to properly describe the steps in really doing a statistical analysis, and because many research studies do not report their primary outcome findings [31] as investigators like to tell good stories from their research after spending say 5 years in doing the study, and *not* reporting on the outcomes that were in the research justification at the time of funding. The hope is that the detailed SAP will give reasons for deviating from the planned analyses to justify the changes between the funded study and the reported study, so readers can take these reasons into account when interpreting the findings from the study. More and more studies are publishing SAPs in advance of conducting the analysis of their data to follow the reasoning process and justifications if there are deviations from the initial analysis plan, and journals are linking the SAP to the protocol and results when searchers are looking for the history of the results papers.

Once the planned data are collected from the participants in the study, the data will be entered into a secure database using codes outlined in a code book, which is being developed, and will guide those making judgements about any coding details including the meaning of the numbers as well as values to display data that are missing for any reason. This will become valuable to those doing the final analysis as they get to understand these codes and take them into account during any database cleaning and statistical analysis. Once entered, and the data will be managed by looking at various descriptive statistics such as sample size, minimum, median, maximum, lower and upper quartiles, mean, standard deviation and interquartile range. Printouts of the raw data and these descriptive statistics will be scanned for anomalies and outliers by comparing the raw data to what is known about the measures. In addition, graphs including plots of distributions of each variable, boxplots and residual plots from models fitted to these data to look for influential data and possible outliers will be created. Any identified unusual data will be checked back through the data collection process to try to see if it should be considered as missing value. These reasons will be documented.

As the randomization has already been implemented, we will practice randomization integrity, taking the plan as it was derived and comparing it to the plan as it was administered in the field to see how it compares. If the randomization is implemented as designed, this will be mentioned as part of the study reports. If there are deviations, these may make the study that is planned as an RCT move more towards that of an observational study, and various analyses will have to be considered for how this should be dealt with and reported as described in the SAP. The primary analysis of the outcome variables will be using intention-to-treat (ITT) as the main method, with others as secondary analyses. Secondary analyses could include randomization as implemented or per protocol, and patterns deemed useful by the investigators. The procedure details will be included in the SAP.

There are three outcome variables being collected from each client in the study: SRQ-20, WHODAS 2.0 and CAGE. The SRQ-20 can be used as individual scores as well as primary of reporting the clients with scores either >7 or <8 to compute a rate for non-case as a proportion, and can be used as the entire interval of scores that are ordinal and so allow for ordinal statistics to be described as well as modeled. The WHODAS 2.0 and CAGE will be analyzed in the form they are scored, with no collapsing to subcategories unless later modeling justifies this. The SAP will describe what might be done in various circumstances.

Since the design of this study is a clustered stepped-wedge design, the descriptive statistics will be displayed in the order of the commune implementation in the wedging: first or second; 3-month intervals over the study; time of observation: baseline, month 1 and month 2, and combining the commune data to up to the district, combining districts up to provinces and finally combining the provinces to the entire country of Vietnam (see Fig. 2). These descriptive statistics will include those listed above: sample size, minimum, median, maximum, lower and upper quartiles, mean, standard deviation and interquartile range, with plots (raw distributions as well as box plots) of the variables by what will be considered as factors: commune, district, province, country, order of implementation and timing of the measurements within each commune. Patterns will be noted to be justified with the statistical analyses conducted later. One type of pattern from the descriptive statistics is to do LOESS smoothing to suggest patterns versus time and the timing may suggest some relationships with time, such as skill of staff within commune or order during the entire trial, that need to be considered in the modeling, as these could impact the assumptions of the stepped-wedge analysis.

Overall, the primary analysis will consider the proportions of non-caseness with the SRQ-20 within commune as the response variable using logistic regression assuming a binomial response distribution as part of the glm (generalized linear model) command in R [32]. The independent variables will be the cluster (commune), order of administration, and baseline value of the response variable, and then timing of the response at months 1 and 2. If the assumptions of the binomial data are not met, then a Poisson or negative-binomial model will be considered next, as they are known to model data better than the binomial. Standard assumptions, such as residual plotting, distributions of the residuals, influence and variance inflation, are checked to see how the models are impacted with the observations for possible deletion and remodeling. Differences between the models will be detected using AIC (Akaike Information Criterion), with smaller values detecting the better models. If some models are very close to each other, these will also be reported. Models that include higher levels of the combined communes as districts, provinces and the entire country will also be explored to gain further insight as the meaning the results. Finally, the raw scores from the SRQ-20 within commune will also be modeled, but differences between this model and the proportions model will defer to the proportions model as it was prespecified as the primary outcome variable in this protocol. In the design, we did not have a study-specific measure of the IC (intraclass correlation), we will estimate it for the four ways of doing the analysis so that future studies can use these estimates in their planning. Results will reported with *p* values in comparison with an alpha value of 0.05, and no correction for multiplicity. In addition, 95% CIs (confidence intervals) will be displayed for the results to make it easier for readers to decide whether the results are clinically important as well as their statistical significance with *p* values. Similar models and methodology will be used with WHODAS 2.0 and CAGE outcome variables. Reports will include all these analyses and any deviations will follow the SAP process for reporting. It is also possible to report all proportions as percentages for a suitable audience to understand.

At the end of the study, we may have some missing data on key variables during the study, even though we have attempted to implement the 18 methods in [33] to minimize missingness in this RCT. If the missingness is overall less than 3%, it will likely not have an impact on the interpretation of our findings [34]. However, with larger than a 3% rate, we will look at various methods for determining the kind of missingness and determine whether the analyses based predominantly on multiple imputation will impact our conclusions, using van Buuren [35] as the main guide and the R package mice (multiple imputation with chained equations) written by the same team as the book [35] which may include sensitivity analyses if these are deemed useful.

The further level of detail will be shown in the SAP to be published later than this protocol and will be be driven by some manuscripts that already have shown how it was done, as well as the various reporting guidelines in EQUATOR for cluster RCTs.

### Data management

Hard copy versions of the questionnaires will be completed by the health and social workers at baseline. Each CHS has a trained health worker who will be responsible for inputting the data into an electronic format using EpiData software [36]. The electronic version will be saved on a secure server. The hard copy questionnaires will be stored in a locked cabinet at the CHS. One- and 2-month assessments will be collected by telephone by outcome assessors based in Hanoi. Data will be similarly entered using EpiData software. Only authorized research team members have will access to hard copy and electronic questionnaires. The hard copy questionnaires and the electronic versions will be saved for 5 years and will then be destroyed, according to Vietnamese requirements. All prescreening data for families that are not recruited to the study will be stored in a locked cabinet in the CHS, and will be destroyed after 1 year, as is standard practice in Vietnam.

### Data monitoring

The Data Monitoring Committee (DMC) will be composed of CHG from Simon Fraser University in Vancouver, a representative from the Institute of Population, Health and Development (PHAD) who is not otherwise involved in the study, and a representative from the Hanoi School of Public Health who is also not involved in the study. The DMC’s mandate will align with the description found in the SPIRIT guidelines [37]. The committee will meet once annually for the duration of project, and will report any concerns to the principal investigators of the study. The DMC is independent from the study sponsor and has no competing interests.

Any important modification to the protocol will be reported by the principal investigators and study team to the research coordinator (JM), who will then communicate them to research ethics boards, the study sponsor, the trial registry and relevant journals.

### Ancillary and post-trial care

While the risks associated with SSM are minimal, it is possible that study participants may experience no improvement in depression symptoms as a result of the intervention, and that they will have a relapse of symptoms post trial, or that they may experience other unexpected outcomes. The involvement of social workers and primary care providers in this study will help to mitigate the risks associated with these circumstances, as the relationship between social workers and patients will not end at the end of the trial period. Social workers will remain in place in the community, and will continue to act as a support to patients and as a bridge between patients and other services. Similarly, this study will increase awareness among community members about the availability of supports for depression in primary care. Patients may continue to access these services after the trial has ended.

## Discussion

This trial plans to assess the effectiveness of a low-cost, community-based intervention for adult depression in eight provinces of Vietnam. Services for depression are very limited in the country, and are currently almost completely unavailable in the primary care context. This trial should provide important evidence about the effectiveness of a SSM intervention that has been validated for use in the Vietnamese context, helping to inform the scale-up of community-based depression services in the country.

This trial takes place in the context of a favorable policy environment, as the government of Vietnam is committed to enhancing community-based services for mental illness, including depression. This study is conducted in partnership with the Vietnamese Ministry of Labor, Invalids and Social Affairs (MOLISA) and with the cooperation of the Ministry of Health. The partnership with the government of Vietnam, and the alignment of this trial with the policy priority to improve community-based mental health services, has several advantages. This trial should produce rigorous data about the effectiveness of the intervention which will be disseminated directly to the ministries and departments of health and social services. This integrated knowledge translation approach hopes to promote evidence-based policy and service development. The involvement of the Government of Vietnam should also help to foster the sustainability and further scale-up of the intervention should the findings be positive. This trial, therefore, has the potential to lead to lasting improvement in access and availability of depression services in Vietnamese communities.

One study limitation is that the trial takes place in eight of Vietnam’s 63 provinces and, therefore, might not be representative of the diversity of the Vietnamese population. Vietnam is home to 54 ethnic groups [38] and there is variation in culture and language between the northern, central and southern regions of the country. Because this trial takes place in provinces in all three regions of the country, however, we are confident that the results could be generalizable.

An additional limitation is that the study measures and tools were designed in Western countries and may, therefore, not be culturally valid for use in Vietnam. We are confident, however, that the work we conducted during the feasibility study, including a review of depression measures that have been validated for use in Vietnam and among Vietnamese populations [25] and the validation of the *Antidepressant Skills Workbook*, support the appropriateness and validity of these measure and tools for use in Vietnam. While the CAGE was not used during the feasibility study, it has been validated for use in many countries, including low-income settings [39] and has been used by Tran et al. [40] in a study of the interaction of perinatal common mental disorders, alcohol use and domestic violence in Vietnam.

## Trial status

At the time of submission, the MAC-FI trial is ongoing, with patient recruitment currently underway.

## Additional file

Additional file 1: SPIRIT Checklist: MAC-FI Study Protocol. (PDF 106 kb)

## Abbreviations

AIC: Akaike Information Criterion
ANOVA: Analysis of variance
CAGE: Cut-down, Annoyed, Guilty, Eye-opener
CHS: Commune Health Station
CI: Confidence intervals
DALYs: Disability-adjusted Life Years
DE: Design Effect
DMC: Data Monitoring Committee
DOLISA: Department of Labor, Invalids and Social Affairs
glm: Generalized linear model
IC: Intraclass correlation
ICC: Intracluster correlation coefficient
ITT: Intention-to-treat
LMIC: Low- and Middle-income country
MOLISA: Ministry of Labor, Invalids and Social Affairs
PHAD: Institute of Population, Health and Development
RCT: Randomized controlled trial
SAP: Statistical Analysis Plan
SRQ: Self-reporting Questionnaire
SSM: Supported Self-management
WHODAS: World Health Organization’s Disability Assessment Scale
